# AKR1B10 promotes breast cancer cell proliferation and migration via the PI3K/AKT/NF-κB signaling pathway

**DOI:** 10.1186/s13578-021-00677-3

**Published:** 2021-08-21

**Authors:** Jiayao Qu, Jia Li, Yaming Zhang, Rongzhang He, Xiangting Liu, Ke Gong, Lili Duan, Weihao Luo, Zheng Hu, Gengsheng Wang, Chenglai Xia, Dixian Luo

**Affiliations:** 1 Department of Laboratory Medicine, Huazhong University of Science and Technology Union Shenzhen Hospital (Nanshan Hospital), Nanshan Avenue, Shenzhou, 518000 Guangdong People’s Republic of China; 2 Translational Medicine Institute, The First People’s Hospital of Chenzhou, University of South China, Hengyang, 423000 Hunan People’s Republic of China; 3 South Medical University Affiliated Maternal & Child Health Hospital of Foshan, Foshan, 528000 Guangdong People’s Republic of China; 4 School of Pharmaceutical Sciences, Southern Medical University, Guangzhou, 520150 Guangdong People’s Republic of China; 5 Center for Laboratory and Pathology, National & Local Joint Engineering Laboratory for High-through Molecular Diagnosis Technology, The First People’s Hospital of Chenzhou, Southern Medical University, Changsha, 423000 Hunan People’s Republic of China; 6 Department of Emergency, The Second Affiliation Hospital, Hunan Normal University, Changsha, Hunan People’s Republic of China

**Keywords:** AKR1B10, PI3K/AKT, NF-κB, Proliferation, Migration, Breast cancer

## Abstract

**Background:**

Aberrant expression of Aldo-Keto reductase family 1 member B10 (AKR1B10) was associated with tumor size and metastasis of breast cancer in our published preliminary studies. However, little is known about the detailed function and underlying molecular mechanism of AKR1B10 in the pathological process of breast cancer.

**Methods:**

The relationship between elevated AKR1B10 expression and the overall survival and disease-free survival of breast cancer patients was analyzed by Kaplan–Meier Plotter database. Breast cancer cell lines overexpressing AKR1B10 (MCF-7/AKR1B10) and breast cancer cell lines with knockdown of AKR1B10 (BT-20/shAKR1B10) were constructed to analyze the impact of AKR1B10 expression on cell proliferation and migration of breast cancer. The expression levels of AKR1B10 were detected and compared in the breast cancer cell lines and tissues by RT-qPCR, western blot and immunohistochemistry. The proliferation of breast cancer cells was monitored by CCK8 cell proliferation assay, and the migration and invasion of breast cancer cells was observed by cell scratch test and transwell assay. The proliferation- and EMT-related proteins including cyclinD1, c-myc, Survivin, Twist, SNAI1, SLUG, ZEB1, E-cadherin, PI3K, p-PI3K, AKT, p-AKT, IKBα, p-IKBα, NF-κB p65, p-NF-κB p65 were detected by western blot in breast cancer cells. MCF-7/AKR1B10 cells were treated with LY294002, a PI3K inhibitor, to consider the impact of AKR1B10 overexpression on the PI3K/AKT/NF-κB signal cascade and the presence of NF-κB p65 in nuclear. In vivo tumor xenograft experiments were used to observe the role of AKR1B10 in breast cancer growth in mice.

**Results:**

AKR1B10 expression was significantly greater in breast cancer tissue compared to paired non-cancerous tissue. The expression of AKR1B10 positively correlated with lymph node metastasis, tumor size, Ki67 expression, and p53 expression, but inversely correlated with overall and disease-free survival rates. Gene Ontology analysis showed that AKR1B10 activity contributes to cell proliferation. Overexpression of AKR1B10 facilitated the proliferation of MCF-7 cells, and induced the migration and invasion of MCF-7 cells in vitro in association with induction of epithelial-mesenchymal transition (EMT). Conversely, knockdown of AKR1B10 inhibited these effects in BT-20 cells. Mechanistically, AKR1B10 activated PI3K, AKT, and NF-κB p65, and induced nuclear translocation of NF-κB p65, and expression of proliferation-related proteins including c-myc, cyclinD1, Survivin, and EMT-related proteins including ZEB1, SLUG, Twist, but downregulated E-cadherin expression in MCF-7 cells. AKR1B10 silencing reduced the phosphorylation of PI3K, AKT, and NF-κB p65, the nuclear translocation of NF-κB p65, and the expression of proliferation- and migration-related proteins in BT-20 cells. LY294002, a PI3K inhibitor, attenuated the phosphorylation of PI3K, AKT, and NF-κB p65, and the nuclear translocation of NF-κB p65. In vivo tumor xenograft experiments confirmed that AKR1B10 promoted breast cancer growth in mice.

**Conclusions:**

AKR1B10 promotes the proliferation, migration and invasion of breast cancer cells via the PI3K/AKT/NF-κB signaling pathway and represents a novel prognostic indicator as well as a potential therapeutic target in breast cancer.

**Supplementary Information:**

The online version contains supplementary material available at 10.1186/s13578-021-00677-3.

## Background

Breast cancer originating from breast epithelial tissue, is one of the most frequently occurring malignant tumors in female adults [1]. Epidemiological studies suggest that the incidence rate of female breast cancer continues to rise annually in China [2]. Despite the significant progress made in diagnosis and treatment, the prognosis for patients remains poor [3]. Better insight into the mechanisms underlying breast cancer would assist with the development of more effective diagnostic and/or therapeutic strategies.

Aldo-Keto reductase family 1 member B10 (AKR1B10), also known as Aldose reductase like protein-1 (ARL-1), is a member of the human aldo-keto reductase (AKR) superfamily which protects cells by reducing aldehyde ketone carbonyl compounds to alcohols [4]. AKR1B10 plays a central role in cancer lipid metabolism by governing the synthesis of lipids through stabilizing acetyl coenzyme-A carboxylase α [5]. In recent studies, AKR1B10 may be involved in the occurrence, and progression of many cancers [6–8]. In patients with hepatocellular carcinoma (HCC), high expression of AKR1B10 is positively correlated with poor prognosis, indicating the protein may effect tumor survival [9]. In patients with breast cancer, elevated AKR1B10 expression is also associated with chemotherapy drug resistance [10]. However, little is known about the detailed function and underlying molecular mechanism of AKR1B10 in the pathology of breast cancer.

In our previous study, we showed that PI3K inhibitors block cell proliferation of breast cancer cells overexpressing AKR1B10, which suggested that AKR1B10 may regulate the AKT signaling pathway in breast cancer. We also noted that AKR1B10-overexpression increased protein level of SNAI1, a transcription factor that regulates EMT. Based on these findings, we comprehensively investigated the effects of AKR1B10 and its associated mechanisms in breast cancer with the goal of developing a novel foundation for future breast cancer diagnosis and treatment.

## Methods

### Patients and tissue specimens

Tumor and adjacent normal tissues were obtained from 33 female breast cancer patients in the First People’s Hospital of Chenzhou between 2013 and 2016. Among them, none of the patients underwent chemotherapy, radiotherapy or immunotherapy prior to surgery. Tumor-Node-Metastasis (TNM) classification was performed according to the 8th edition of the American Joint Committee on Cancer (AJCC) staging system. The study was approved by the Ethics Committee of The First People’s Hospital of Chenzhou and informed consent was obtained from all patients.

### Cell culture and treatments

The human breast cancer cell lines MCF-7 (RRID:CVCL_0031) and BT-20 (RRID:CVCL_0178) were purchased from the Shanghai Institute of Biochemistry and Cell Biology, Chinese Academy of Sciences (Shanghai, China). All human cell lines have been authenticated using short-tandem repeats (STR) profiling within the last 3 years. All experiments were performed with mycoplasma-free cells. Overexpression stable cell line and knockdown stable cell line were established as previously described in our laboratory [7]. For knockdown, 5 × 10^4^ BT-20 cells were planted on a 6-well plates. When the cells have grown to 70–80% confluence, replace fresh RPMI1640 medium without fetal bovine serum and use the Scramble or shAKR1B10 virus infects the cells. After culturing for 72 h, the stable BT-20/Scramble and BT-20/shAKR1B10 cells were obtained by puromycin screening. The shAKR1B10 sequence was as following: shAKR1B10#1: GATCCGTGTTGCAATCCTCTCATTTTCAAGAGAAATGAGAGGATTGCAACATTTTTTG. shAKR1B10#2: GATCCGAAGTGAAAGAAGCAGTGAATCAAGAGTTCACTGCTTCTTTCACTTTTTTTTG. Wherever mentioned, the cells were treated with 30 µM and/or 50 µM of the PI3K inhibitor LY294002 (CST) for 48 h.

### Immunohistochemistry (IHC)

IHC staining was conducted as previously described [11]. A rabbit polyclonal antibody against AKR1B10 (1:100, self-prepared) was used to detect AKR1B10. The IHC score was calculated by multiplying the percentage of positive cells with the intensity of staining. The intensity of IHC staining was designated as: 0 (no staining), 1 (weak staining), 2 (moderate staining), and 3 (strong staining). The percentage of stained cells was determined as: 1 (1–25%), 2 (26–50%), 3 (51–75%), and 4 (76–100%). The IHC score of AKR1B10 expression was evaluated by two experienced pathologist.

### Quantitative real-time RT-PCR

Total RNA was extracted from cell lysates and breast normal/tumor tissues using RNA TRIzol Reagent (Invitrogen, USA) according to manufacturer’s instructions. Reverse transcription was performed using the GoScript Reverse Transcription System (Promega, USA) following manufacturer’s protocols. RT-PCR reactions were performed using SYBR Premix Ex Taq II (Takara, Japan) in a Light Cycle480 Real-Time PCR Detection System (Roche, Germany) following the manufacturer’s instructions. The relative mRNA levels were normalized against GAPDH using the 2^−ΔΔCt^ formula. The primer sequences were as follows:

- GAPDH forward: ACCACAGTCCATGCCATCAC;
- GAPDH reverse: ACCACCCTGTTGCTGTA;
- AKR1B10 forward: GCTGAGCTATCTGGACGTCT;
- AKR1B10 reverse: CGTTACAGGCCCTCCAGTTT.

### Cell proliferation assay

Cell Counting Kit-8 (CCK-8, Beyotime, China) was used to detect cell proliferation. Briefly, 1 × 10^3^ breast cancer cells were seeded in each well of a 96-well plate and a CCK-8 kit was used every 24 h. The absorbance was measured 24 h later at 450 nm. The cell growth curve was generated over 3 consecutive days using the CCK-8 assay.

### Wound healing assay

Cells were seeded and cultured in six-well plates for 24 h. Wounds were created by introducing scratches in the monolayer of cells using 100 µL pipette tips. The medium was then replaced with serum-free medium. Plates were washed twice with fresh medium to remove non-adherent cells after the cells had been cultured for 48 h, and then photographed. Finally, the distances between wound edges were measured. The rate of wound healing = [(the wound width of 0 h − the wound width of 24 or 48 h)/the wound width of 0 h] × 100%.

### Migration and invasion experiments

Cell migration and invasion were measured using a Corning polycarbonate film insert transwell chamber (Corning, USA) containing 8 μm pores in the presence or absence of Matrigel (BD Biosciences, USA) according to the manufacturer’s protocol. 5 × 10^4^ cells per well were seeded in the upper of chamber without Martrigel for migration assay, and 1 × 10^5^ cells per well were seeded in the upper of chamber with Martrigel for invasion assay. The medium containing 10% FBS was placed in the lower chamber as an attractant. 24 h later, the cells were fixed in 4% paraformaldehyde for 20 min and then stained with 0.1% crystal violet for 10 min. The cells in the upper chamber were removed with a cotton swab and dried, and the images were captured in 6 random zones under each microscope. The number of cells was counted by Image software. The mean cell count of three independent membranes was defined as the migration or invasion index. The experiment was repeated three times.

### Bioinformatics analysis

The Kaplan Meier-plotter database (http://www.kmplot.com) was used to evaluate the relationship between AKR1B10 expression and overall or disease-free survival rates in patients with breast cancer. Coexpedia (http://www.coexpedia.org) [12] was used for AKR1B10 Gene Ontology (GO) term analysis.

### Western blotting

Western blotting analyses were performed as previously described [13]. In detail, total protein was extracted using RIPA buffer, quantified by BCA assay, and subjected to SDS-PAGE gel electrophoresis and transferred to nitrocellulose membrane (NC). The blot was blocked with 5% non-fat milk and then incubated with primary antibodies overnight at 4 °C and then incubated with the secondary antibody for 1.5 h on a room temperature. The primary antibodies used in this study are listed in Additional file 1: Table S1. After extensive washing in PBST, the expression levels of the proteins were detected by Quantity-one software (Bio-Rad Laboratories, USA) using the ECL-chemiluminescent kit. The signal of protein bands was quantified by ImageJ software for Windows (NIH, USA).

### PIP_3_ ELISA assay

PIP_3_ concentrations were measured using the enzyme-linked immunosorbent assay by ELISA kit (Jianglai Bio, China). Cells were rinsed with PBS, detached by scraping, collected by centrifugation, and disrupted by ultrasonic treatment. The blank well, standard well and the sample well were set, respectively; 40 µL sample dilution buffer and 10 µL sample solution were added in the test well. 100 µL of enzyme labeling reagent was added to each well, except for blank wells. After incubation for 30 min, absorbance was measured at 450 nm and PIP_3_ concentration was calculated using a standard curve.

### In vivo tumor xenograft experiments

Animal experiments were performed on female BALB/c nude mice. At 6 weeks of age, the mice were inoculated s.c. with 5 × 10^6^ MCF-7/Vector or MCF-7/AKR1B10 cells mixed 1:1 with Matrigel (BD Biosciences Pharmingen) (n = 6 mice per group). The Mice receiving MCF-7 cells were, before tumor cell inoculation, implanted s.c. with a slow release estradiol pellet (0.72 mg, 60 days, Innovative Research of America). Additionally, 5 × 10^6^ BT-20/Scramble cells or BT-20/shAKR1B10 cells were injected into the mammary fat pad of the 2 groups respectively (n = 6 mice per group). Four weeks later, the mice were sacrificed, and the xenograft tumor tissues were weighed. The study protocol complied with the ARRIVE guidelines and was carried out by following the National Institutes of Health guide for the care and use of laboratory animals.

### Statistical analysis

Statistical Package for the Social Sciences (SPSS) V.20 for Windows was used to run statistical analyses. Student’s t tests and ANOVA were used to compare the statistical differences between groups. A p-value less than 0.05 was considered to indicate a statistically significant difference.

## Results

### AKR1B10 expression in breast cancer tissues is associated with clinicopathological features

**Fig. 1:**
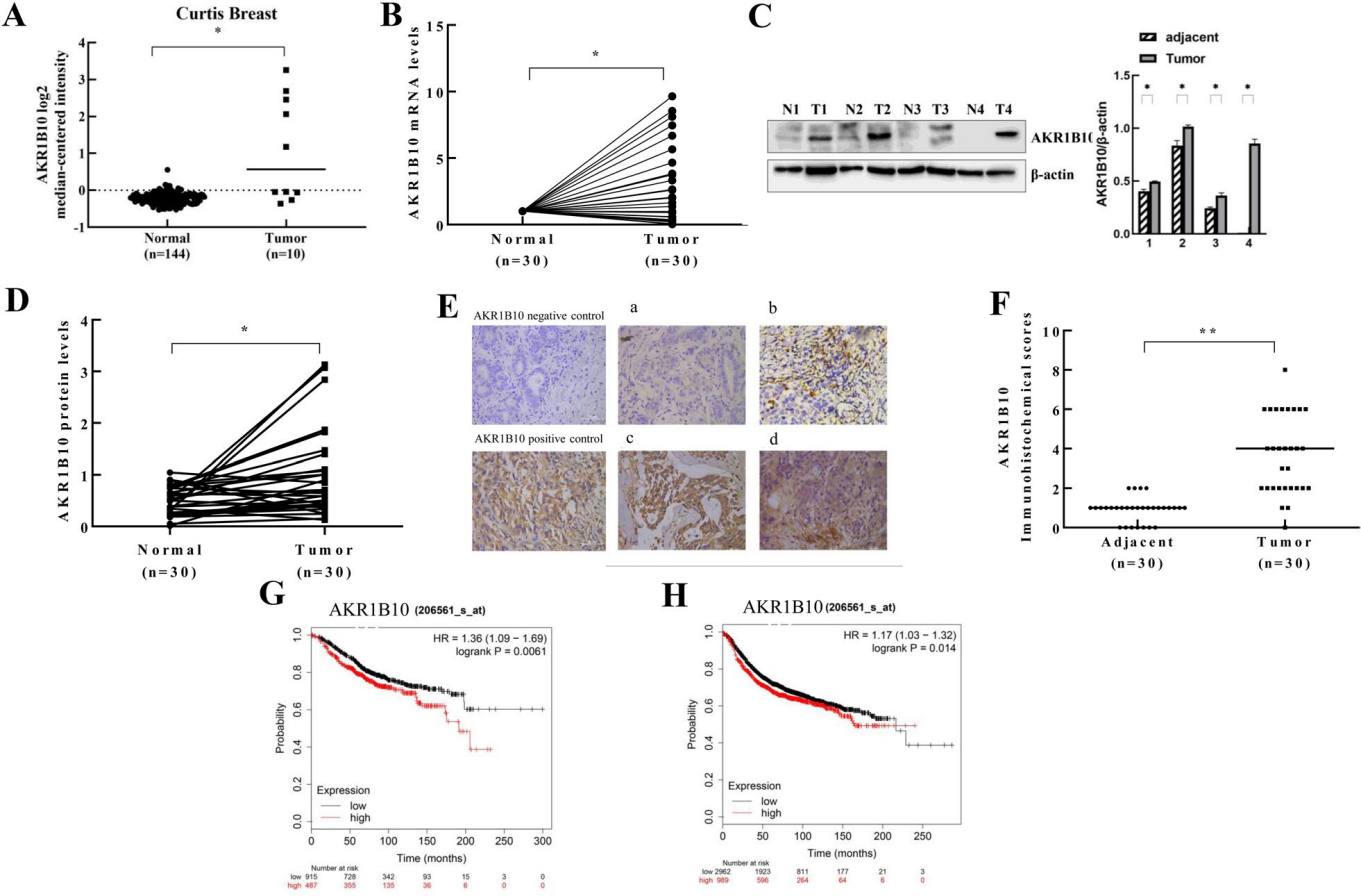
AKR1B10 is highly expressed in breast cancer tissues and correlates with patient prognosis. **A** AKR1B10 mRNA was upregulated in cancerous breast tissue compared to normal tissue in Curtis Breast dataset; **B** AKR1B10 mRNA levels was greater in 8 fresh breast tumor specimens than in paired adjacent normal tissue. The AKR1B10 relative expression levels were normalized to 1 in adjacent normal tissue; **C**, **D** AKR1B10 protein levels were higher in 4 fresh breast cancer tissues than in adjacent normal tissues. Data quantification relative to β-actin levels were compared for AKR1B10. **E**, **F** The immunohistochemical staining (IHC) score of AKR1B10 in cancerous tissue was significantly higher than the scores in adjacent normal tissues. Images **a**–**d** are representative images of 4 levels of expression from 0 to 8 score, such as 0 for no staining (**a**), 2 for week staining (**b**), 4 for moderate staining (**c**), 6 for strong staining (**d**). Scale bar = 50 μm; **G**, **H** Kaplan–Meier estimation revealed significantly lower overall and disease-free survival rates in patients with AKR1B10^high^ breast cancer than that in AKR1B10^low^ patients; all experiments were performed in triplicate and a representative WB analysis is depicted

To investigate the expression of AKRB10 in breast cancer, the Curtis breast dataset data including 10 cases of ductal carcinoma in situ and 144 cases of normal breast tissue were obtained from the Oncomine database. AKR1B10 mRNA levels were significantly higher in breast cancer tissues compared to normal tissues (Fig. 1A). We analyzed AKR1B10 mRNA and protein levels in 30 pairs of fresh breast cancer tissue/normal tissue by qRT-PCR and western blotting. Both mRNA and protein levels of AKR1B10 were significantly higher in tumor breast tissues than adjacent non-tumor counterparts (Fig. 1B–D). Furthermore, immunohistochemistry (IHC) was used to examine AKR1B10 expression in 30 breast cancer samples and paired adjacent non-tumor tissue. AKR1B10-positive staining was significantly higher in cancer tissues than normal breast tissues (Fig. 1E, F). These results demonstrate that AKR1B10 is highly expressed in breast cancer tissues.

Next, the association between AKR1B10 expression and clinicopathological characteristics of patients with breast cancer (n = 63) was analyzed. The protein levels of AKR1B10 distinctly correlated with lymph node metastasis (p = 0.016), tumor size (p = 0.010), Ki67 expression (p = 0.036), and p53 expression (p = 2.73E−04) (Table 1). Kaplan–Meier survival analysis showed that AKR1B10^high^ patients had markedly lower overall survival (OS, p = 0.0061) and disease-free survival (DFS, p = 0.014) rates compared to AKR1B10^low^ breast cancer patients (Fig. 1G, H). Taken together, these results indicate that AKR1B10 may play an important role in breast cancer development and pathology.

**Table 1:** The relationship between AKR1B10 expression and clinicopathological features of patients with breast cancer

Clinical parameters	n	AKR1B10 levels (IHC)		X^2^	R	P
		≤ 4	> 4			
Age						
< 50	34	23	11	1.035	0.435	0.317
≥ 50	29	16	13			
LN metastasis						
Yes	38	19	19	5.755	0.302	0.016
No	25	20	5			
Tumor diameter (cm)						
≤ 2	20	17	3	6.628	0.320	0.010
> 2	43	22	21			
Ki67 (%)						
≤ 20	29	22	7	4.439	0.265	0.036
> 20	34	17	17			
P53 (%)						
≤ 40	29	25	4	13.457	0.462	2.73E−04
> 40	34	14	20			
ER						
+	31	21	10	0.882	0.118	0.356
−	32	18	14			
PR						
+	25	17	8	0.653	0.102	0.427
−	38	22	16			
ERBB2						
+	45	27	18	0.242	− 0.062	0.629
−	18	12	6			

### AKR1B10 promotes proliferation, migration, invasion of breast cancer cells

**Fig. 2:**
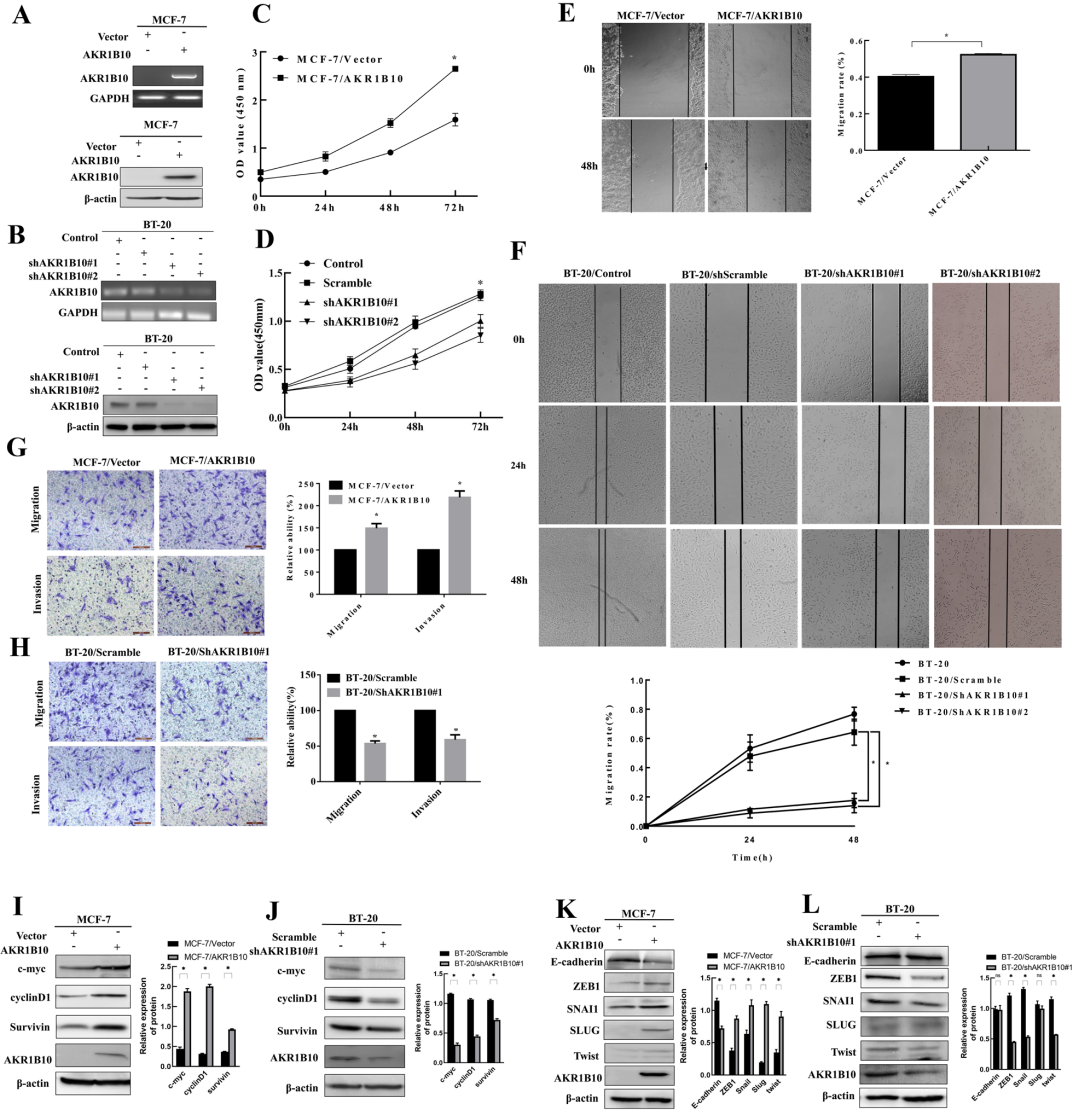
AKR1B10 promotes the proliferation, migration and invasion of breast cancer cells in vitro via regulation of epithelial-mesenchymal transition (EMT) and proliferation-related genes. **A**, **B** RT-PCR and western blot analysis of gene expression changes upon AKR1B10 overexpression and knockdown in MCF-7 cells and BT-20 cells, respectively; **C**, **D** AKR1B10 overexpression increased cell proliferation in MCF-7 cells and AKR1B10 knockdown inhibited cell proliferation in BT-20 cells; **E**, **F** MCF-7 cell migration was significantly enhanced when AKR1B10 was overexpressed while BT-20 cell migration was significantly inhibited when AKR1B10 was knocked down in a scratch wound assay; **G** Migration and invasion ability of MCF-7 cells after AKR1B10 overexpressing in a transwell assay. **H** Migration and invasion ability of BT-20 cells after AKR1B10 knocking down in a transwell assay. **I**, **J** Western blotting analysis showed increased protein levels of c-myc, cyclinD1, and Survivin in MCF-7/AKR1B10 cells, and reduced protein levels in BT-20/shAKR1B10#1 cells, respectively; **K** Western blotting analysis showed significantly upregulated ZEB1, SNAI1, SLUG, Twist and downregulated E-cadherin in MCF-7/AKR1B10 cells compared to vector control cells; **L** Western blotting analysis showed significantly reduced ZEB1, Twist at protein level in BT-20/shAKR1B10#1 cells compared to scramble control cells. Experiment was repeated three times. Representative results were presented. *P < 0.05

To further study the biological function of AKR1B10 in breast cancer, we first established stable breast cancer cell lines overexpressing AKR1B10 in MCF-7 cells by lentiviral infection. Similarly, BT-20 cells with stable knockdown of AKR1B10 were established by lentiviral-mediated expression of shRNA. Stable overexpression and knockdown of the protein was confirmed by RT-PCR and western blotting analysis (Fig. 2A, B). The clinicopathological correlation analysis indicates a role for AKR1B10 in the proliferation and metastasis of breast cancer cells. We thus examined functional terms correlated with AKR1B10 genes using Gene Ontology (GO) terms in the coexpedia database. Gene function analysis of AKR1B10 indicated AKR1B10 is significantly associated with the terms “cell cycle process”, “regulation of cell proliferation”, ”regulation of cell cycle” etc. (Table 2). This further suggested that AKR1B10 may be associated with the tumor progression. Next, we performed CCK8 proliferation assays, scratch wound assays and transwell assays. CCK8 assays showed that overexpression of AKR1B10 promoted cell proliferation of MCF-7 cells compared to the MCF-7/Vector, while knockdown reduced cell proliferation of BT-20 cells compared to the BT-20/Scramble (Fig. 2C, D). AKR1B10 overexpression in MCF-7 cells significantly enhanced cell migration and invasion (Fig. 2E, G). Conversely, loss of AKR1B10 suppressed cell migration and invasion ability (Fig. 2F, H). These in vitro results demonstrated that AKR1B10 not only affects cell proliferation but also promotes migration and invasion in breast cancer cells.

**Table 2:** AKR1B10 gene ontology functional analysis

GO enrichment	Term		P
Biological process	GO:0007049	Cell cycle	0.01653
Biological process	GO:0042127	Regulation of cell proliferation	0.03943
Biological process	GO:0051726	Regulation of cell cycle	0.04398
Biological process	GO:0000083	Regulation of transcription involved in G1/S transition of mitotic cell cycle	0.04766

Studies have shown that proliferation of cancer cells largely relies on proliferation-related proteins including c-myc, Survivin and cyclinD1, which prompted us to investigate whether these regulators were involved in AKR1B10-induced proliferation of breast cancer cells. As expected, c-myc, Survivin and cyclinD1 were downregulated in BT-20 cells/shAKR1B10, but upregulated in MCF-7 cells/AKR1B10 (Fig. 2I, J). It is well known that several Epithelial–mesenchymal transition (EMT)-related proteins play important roles in cell migration and invasion during tumor progression. We examined changes in the expression levels of the EMT-related proteins ZEB1, SNAI1, SLUG, Twist and E-cadherin after AKR1B10-gene intervention in the breast cancer cells by western blot. ZEB1, SNAI1, SLUG, Twist expression increased, and E-cadherin expression decreased following AKR1B10 overexpression in MCF-7 cells, whereas ZEB1 and Twist were downregulated following AKR1B10 knockdown in BT-20 cells (Fig. 2K, L). These data suggests that AKR1B10-induced proliferation, migration and invasion observed in breast cancer cells is mediated by regulation of transcription factors that control cell proliferation and EMT.

### AKR1B10 activates the NF-κB pathway

Previous studies have shown that NF-κB signaling plays a critical role in tumor cell progression [14]. Hence, we examined the phosphorylation status of both IKBα and NF-κB p65 in breast cancer cells with AKR1B10 knockdown or overexpression.

**Fig. 3:**
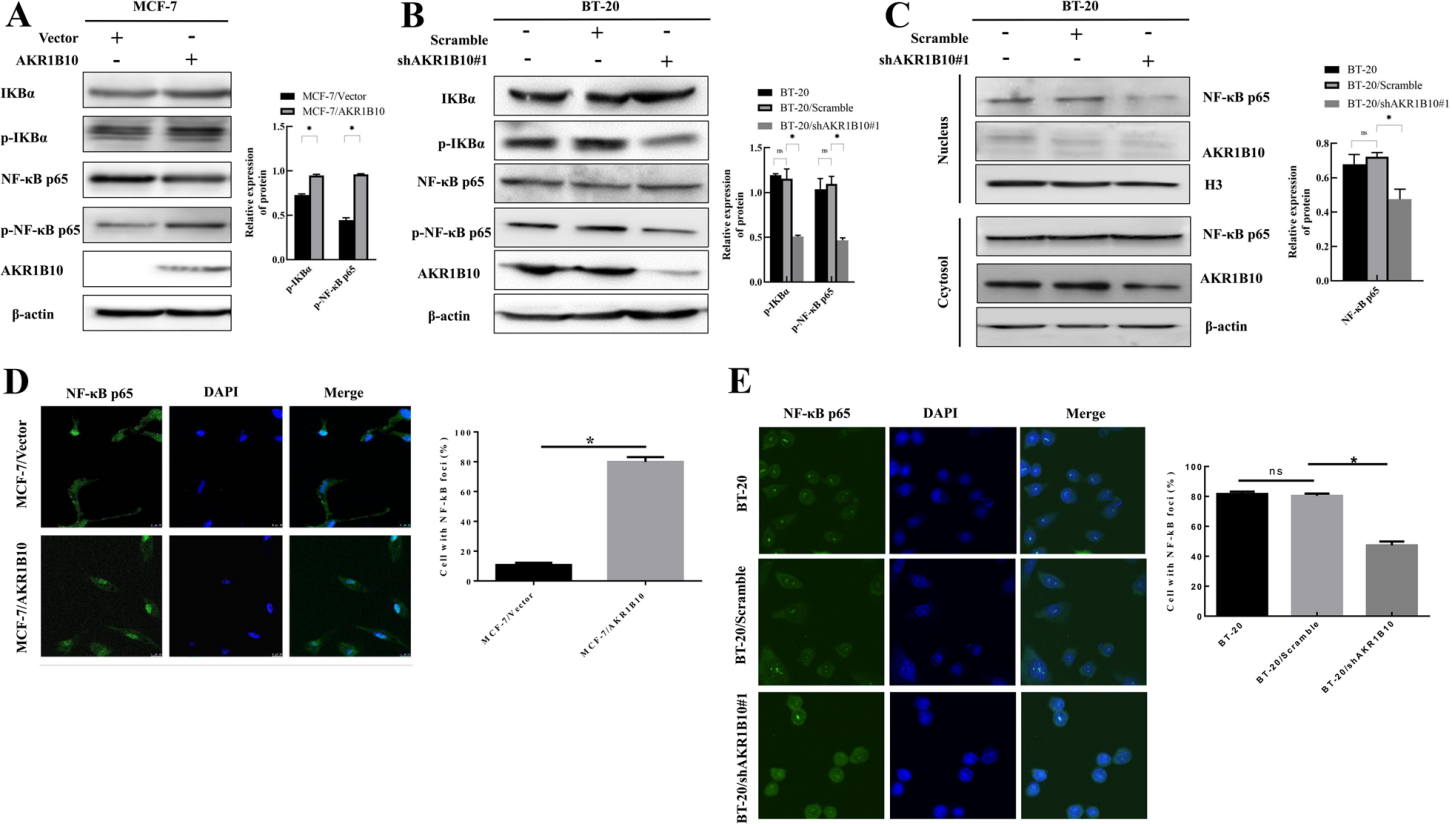
AKR1B10 activates the NF-κB pathway in breast cancer cells. **A**, **B** Western blotting analysis showed increased protein levels of Phosphorylated-IKBα and Phosphorylated-NF-κB p65 in MCF-7/AKR1B10 cells, and reduced protein levels of Phosphorylated-IKBα and Phosphorylated-NF-κB p65 in BT-20 cells/shAKR1B10#1 cells, respectively; **C** Western blotting analysis showed decreased protein levels of nuclear NF-κB p65 in nucleus in BT-20/shAKR1B10#1 cells; **D** immunofluorescence colocalization experiments showed enhanced nuclear translocation of NF-κB p65 in MCF-7/AKR1B10 cells compared to vector control cells. **E** Immunofluorescence colocalization experiments showed decreased nuclear translocation of NF-κB p65 in BT20/shAKR1B10#1 cells compared to scramble control cells. Experiment was repeated three times. Representative results were presented. *P < 0.05; **P < 0.01

Overexpression of AKR1B10 significantly increased the phosphorylation of IKBα and NF-κB p65 compared to control cells (Fig. 3A), while knockdown of AKR1B10 inhibited the phosphorylation of these molecules (Fig. 3B). We also examined the nuclear expression of NF-κB p65 by western blotting in AKR1B10-knockdown BT-20 cells as NF-κB p65 is a known nuclear transcription factor which enters the nucleus upon activation. As shown in Fig. 3C, knockdown of AKR1B10 reduced NF-κB p65 protein presence within the nucleus. This result was supported by immunofluorescence analysis, which revealed increased nuclear accumulation of NF-κB p65 in AKR1B10-overexpressed MCF-7 cells (Fig. 3D), but decreased nuclear accumulation of NF-κB p65 in AKR1B10-knockdown BT-20 cells (Fig. 3E), Together, these results suggest that increased levels of AKR1B10 activates the NF-κB signaling pathway.

### AKR1B10 activates PI3K/AKT pathway

**Fig. 4:**
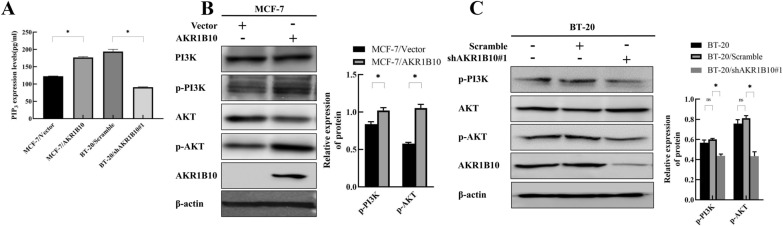
AKR1B10 promotes PIP_3_ expression and subsequent activation of the PI3K/AKT pathway in breast cancer cells. **A** PIP_3_ ELISA kit analysis showed increased expression levels of PIP_3_ in MCF-7/AKR1B10 cells, and reduced expression levels of PIP_3_ in BT-20/shAKR1B10#1 cells, respectively; **B**, **C** Western blotting analysis showed increased protein levels of Phosphorylated-PI3K and Phosphorylated-AKT in MCF-7/AKR1B10 cells, and reduced protein levels of Phosphorylated-PI3K and Phosphorylated-AKT in BT-20/shAKR1B10#1 cells, respectively. Experiment was repeated three times. Representative results were presented. *P < 0.05

Recently, AKR1B10 has been reported to regulate phosphatidylinositol (3,4)-bisphosphate (PIP_2_) production in breast cancer cells [5]. PIP_2_ is the substrate of phosphatidylinositol 3-kinase (PI3K) [15]. PIP_3_ is produced from PIP_2_ by activated PI3K [15]. Gene function analysis indicated that AKR1B10 was significantly associated with the terms “positive regulation of protein kinase B signaling” (data not shown). Based on these results, we first analyzed the levels of PIP_3_ in breast cancer cells. As shown in Fig. 4A, overexpression of AKR1B10 increased PIP_3_ levels in MCF-7 cells, while knockdown of AKR1B10 decreased PIP_3_ levels in BT-20 cells. These results prompt that AKR1B10 may be involved in regulating PIP_3_ synthesis. Next, we analyzed the phosphorylation and subsequent activation of PI3K/AKT signaling pathway-related proteins. AKR1B10 overexpression elevated the levels of phosphorylated-PI3K and phosphorylated-AKT, and not surprisingly, inhibition of AKR1B10 decreased the levels of these molecules in breast cancer cells (Fig. 4B, C). These findings suggest that the PI3K/AKT signaling pathway participates in AKR1B10-induced pathological progression observed in breast cancer cells.

### PI3K/AKT-activated NF-κB signaling pathway contributes to the functionality of AKR1B10 in breast cancer

**Fig. 5:**
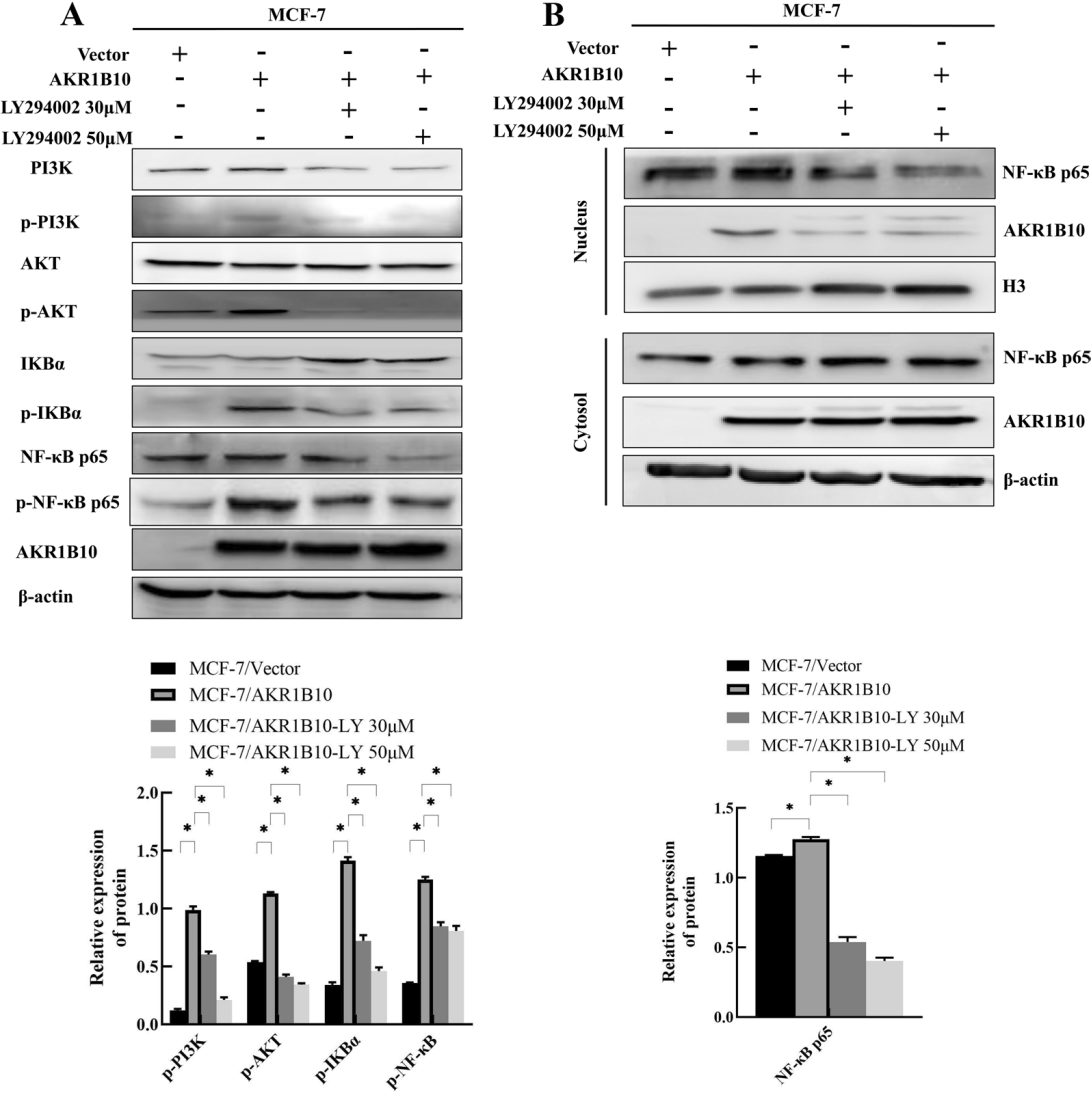
Nuclear translocation of NF-κB p65 was inhibited by PI3K inhibition. **A** Western blotting analysis showed that treatment with LY294002 (30 µM, 50 µM) attenuated AKR1B10-induced Phosphorylated-PI3K, Phosphorylated-AKT; Phosphorylated-IKBα in MCF-7/AKR1B10 cells; **B** Western blotting analysis showed that treatment with LY294002 (30 µM, 50 µM) attenuated AKR1B10-incuded expression of nuclear NF-κB p65. Experiment was repeated three times. Representative results were presented. *P < 0.05

Previous studies have shown that NF-κB expression is regulated by inhibitory IκB proteins, which can be controlled by the upstream PI3K/AKT signaling pathway. To test whether up-regulation in NF-κB p65 protein expression was due to activation of the PI3K/AKT signaling pathway, we used LY294002, a PI3K specific inhibitor to block this pathway. MCF-7/AKR1B10 cells were treated with 30 µM and 50 µM PI3K inhibitor for 48 h. As anticipated, the levels of phosphorylated-PI3K and phosphorylated-AKT were significantly reduced in the presence of PI3K inhibitor (Fig. 5A). Interestingly, we found that LY294002 dampened the levels of phosphorylated-IKBα and phosphorylated-NF-κB p65 in MCF-7/AKR1B10 cells (Fig. 5A). However, the changes in phosphorylated-NF-κB p65 were not concomitant with a decrease in NF-κB p65 protein levels. We further evaluated the effects of PI3K inhibitor on NF-κB p65 nuclear translocation using a nuclear/cytosol fractionation assay. Nuclear expression of NF-κB p65 was decreased after treatment with the PI3K inhibitor in MCF-7/AKR1B10 cells (Fig. 5B). Taken together, these results demonstrate that AKR1B10 activates the NF-κB signaling pathway, which can be inhibited by PI3K specific inhibitor LY294002. AKR1B10 may thus promote breast cancer progression by activating the PI3K/AKT/NF-κB signaling cascade.

### AKR1B10 promotes breast cancer growth in vivo

**Fig. 6:**
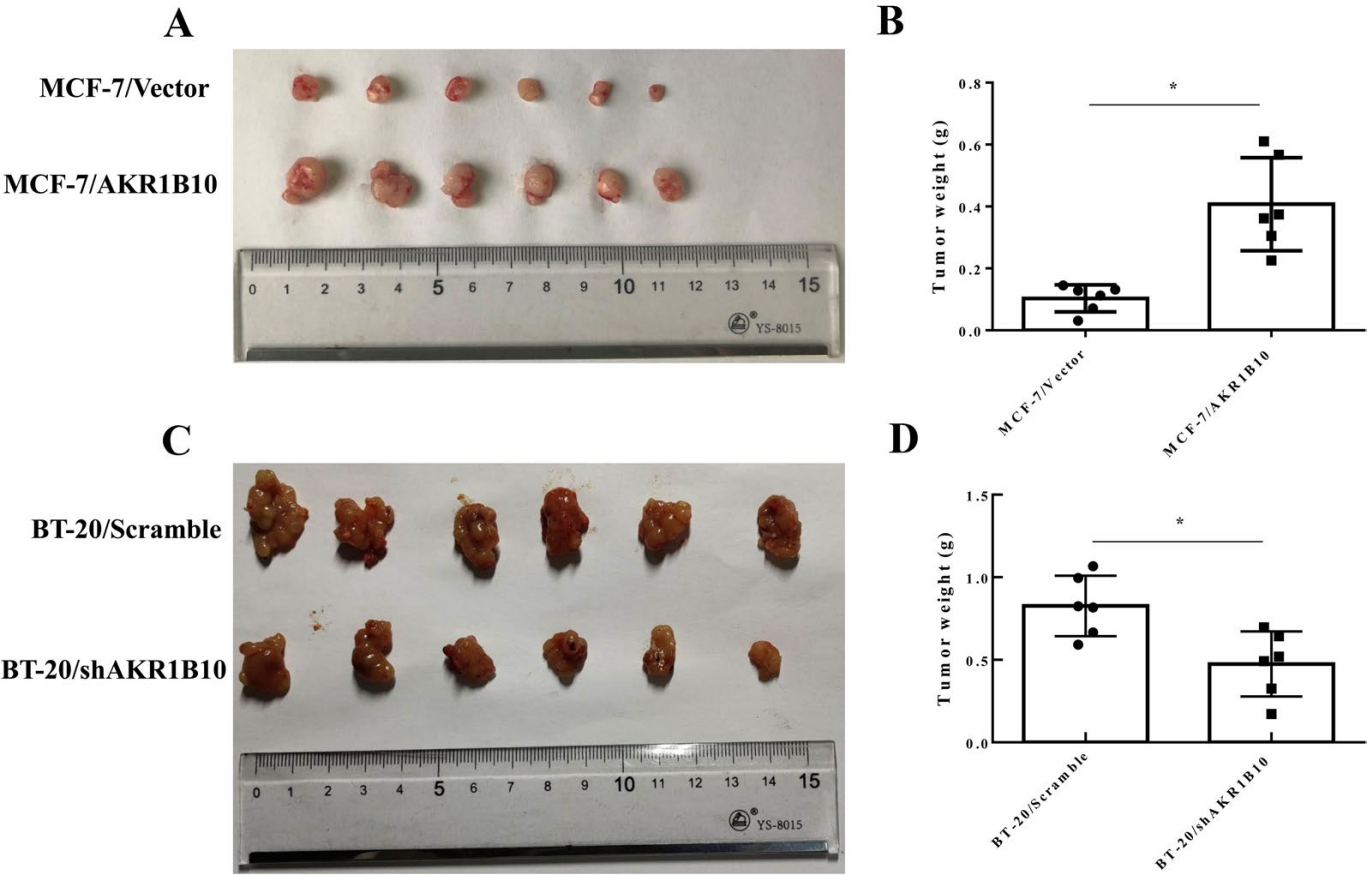
AKR1B10 promotes breast cancer growth in vivo. **A**, **B** AKR1B10 overexpressed promoted the growth of MCF-7 cells in vivo, compared to vector control-injected cells. **C**, **D** AKR1B10 knockdown inhibited the growth of BT-20 cells in vivo, compared to scramble control-injected cells

We investigated the effect of AKR1B10 on the growth of breast cancer in an in vivo mouse xenograft model. After implanting s.c. with a slow release estradiol pellet, the mice were inoculated s.c. with 5 × 10^6^ MCF-7/Vector or MCF-7/AKR1B10 cells. BT-20/Scramble and BT-20/shAKR1B10 breast cancer cells were implanted orthotopically into nude mice mammary fat pads. About four weeks after inoculation, the nude mice were sacrificed, and tumors were collected and weighted. As shown in Fig. 6, consistent with the results of the CCK8 assay, overexpression AKR1B10 significantly promoted tumor growth, while loss of AKR1B10 inhibited tumor growth in vivo. This suggests that high expression levels of AKR1B10 in tumors are closely related to the proliferation of breast cancer cells.

## Discussion

Breast carcinoma is the most common malignant tumor in females [1, 16]. According to statistics in China, breast cancer accounts for 17% of all malignant tumors in women [2]. Early diagnosis and treatment strategies for breast cancer have made significant progress, but the mortality rate of breast cancer is still high, mainly due to breast cancer metastasis [17]. The processes driving breast cancer progression are complex and further research on the underlying molecular mechanisms is warranted.

As a member of aldehyde ketone reductase superfamily, AKR1B10 was first studied in the oncogenic mechanisms of lung cancer [18]. A few studies have shown that AKR1B10 is closely correlated with the progression of cancer. In oral squamous cell carcinoma [8, 19], non-small cell lung carcinoma [20] and liver carcinoma [6], high expression levels of AKR1B10 in patients is correlated with poor prognosis. Large-scale and multicenter studies have demonstrated that AKR1B10 may serve as a serological marker for HCC in humans [9]. AKR1B10 also plays a critical role in invasion and chemoresistance in breast cancer cells [10]. In our study, we found that AKR1B10 is highly expressed in breast cancer tissue. Elevated expression of AKR1B10 is correlated with clinicopathological features (e.g. tumor size, Ki67 expression, and p53 expression), negative overall survival rate and negative disease-free survival rate. Similar findings were also reported in another related study [11]. These results supported the critical role of AKR1B10 in breast cancer.

EMT is a biological process whereby epithelial-like cells transform into mesenchymal-like cells and gain migratory and metastatic potential [21]. EMT plays an important role during development as it essential for the formation of the gastrointestinal system and neural tube formation, as well as in the wound-healing process [22]. Malignant progression of primary tumors highly depend on EMT to promote cellular dispersion throughout the body [23–25]. In the present study, we show that overexpression of AKR1B10 enhances migration and invasion of breast cancer cells in vitro, while knockdown decreases cell dispersion. The increased migratory potential could be attributed to a decrease in E-cadherin expression upon AKR1B10 overexpression in MCF-7 cells. Further, we show that AKR1B10 influences the expression of EMT-related transcription factors, including ZEB1, SNAI1, SLUG and Twist. These results demonstrate possible roles for AKR1B10 in the regulation of EMT. However, it should be noted that knockdown of AKR1B10 in BT-20 cells did not result in significant changes in all EMT-related transcription factors. Taking into account the heterogeneity of tumor cells, some differences in the activation of EMT induced by AKR1B10 can be expected. Abnormal proliferation is one of the hallmarks of tumorigenesis. Cell growth-related factors, including c-myc, cyclinD1 and Survivin, constitute an important group of molecules that regulate cell proliferation in tumor cells [26]. In our study, we found that elevated expression of AKR1B10 in MCF-7 cells upregulates cyclinD1, c-Myc and Survivin, while knockdown of AKR1B10 in BT-20 cells downregulates these molecules. Taken together, we show that AKR1B10 regulates different genes known to regulate breast cancer proliferation, migration and invasion.

The PI3K/AKT signaling pathway is involved in various cellular processes such as glucose metabolism, apoptosis, cell proliferation, and cell migration [15, 27, 28]. Sustained activation of EMT has been reported to be regulated by the PI3K/AKT signaling pathway in numerous cancers. For example, cPLA2α has been shown to mediate EMT via the PI3K/AKT pathway [29]. Previous studies suggest that PIP_2_ is upregulated in breast cancer cells in which AKR1B10 is overexpressed [5], which prompted us to investigate the phosphatidylinositol and PI3K/AKT pathway in detail. Consistently, our study demonstrated that the PI3K/AKT pathway was upregulated in AKR1B10-induced breast cancer progression. Further, studies have shown that activation of the PI3K/AKT pathway is also involved in activation of NF-κB via phosphorylation of inhibitory IκBα [30]. Our study found that LY294002, a specific PI3K inhibitor, suppressed NF-κBp65 nuclear translocation, which indicates that the activation of the PI3K/AKT/NF-κB signaling cascade is regulated by AKR1B10. CyclinD1, c-myc, Survivin and EMT-related proteins are downstream intracellular signal molecules for NF-κB signaling [26, 31]. AKR1B10 may therefore regulate proliferation-related and EMT-related proteins via the PI3K/AKT/NF-κB signaling cascade. Another recent study found that AKR1B10 can promote proliferation and migration/invasion of breast cancer cells via the ERK and FAK/Src/Rac1 signaling pathway [7, 32]. Several studies have also demonstrated that PI3K/AKT and MEK/ERK signaling pathways can be activated together in tumors [33–35]. However, we noted a report that AKRB10 may play an important role in metabolic adaptability [36]. Some results were inconsistent with our results. However, considering that different cells were used, AKR1B10 may have different mechanisms in different cells. Taken together, AKR1B10 may affect several signaling pathways related to cell proliferation and migration in breast cancer. There is also evidence of elevated induction of AKR1B10 in some healthy tissues, such as colon tissue [4], suggesting that AKR1B10 regulation of cell proliferation and migration may be context and/or tissue dependent.

## Conclusions

Our study demonstrates that elevated expression levels of AKR1B10 in breast cancer tissues correlates with lymph node metastasis, tumor size and poor prognosis. AKR1B10, as a critical onco-protein, may activate the PI3K/AKT/NF-κB signaling cascade to promote the expression of proliferation-related and EMT-related proteins and consequently stimulate proliferation, migration and invasion in breast cancer cells. Thus, AKR1B10 might serve as a new prognostic indicator and a potential therapeutic target for breast cancer.

## Supplementary Information

**Additional file 1: Table S1.** Primary antibodies used in this study.

## Data Availability

All the data and material could be traced from the paper or can be requested from the corresponding author.
