# Supplementary material for: AKR1B10 promotes breast cancer cell proliferation and migration via the PI3K/AKT/NF-κB signaling pathway
Source: Cell Biosci. 2021 Aug 21;11:163. doi: 10.1186/s13578-021-00677-3 (PMC8379827; doi:10.1186/s13578-021-00677-3)
Supplement: Supplementary file 1 — Additional file 1: Table S1. Primary antibodies used in this study. [file 13578_2021_677_MOESM1_ESM.doc]

| **Table S1:** Primary antibodies used in this study | | |  |
| --- | --- | --- | --- |
| Target | Dilution | Company | Catalog Number |
| AKR1B10 | 1:500 | Self-prepare | - |
| c-myc | 1:1000 | CST | 18583 |
| CyclinD1 | 1:1000 | CST | 55506 |
| survivin | 1:1000 | Beyotime | AF1222 |
| E-cadherin | 1:1000 | abcam | ab1416 |
| ZEB1 | 1:1000 | abcam | ab228986 |
| Snail | 1:1000 | abcam | ab229701 |
| Slug | 1:1000 | abcam | ab51772 |
| twist | 1:1000 | abcam | ab175430 |
| NF-κB p65 | 1:1000 | CST | 8242 |
| phospho-NF-κB p65 | 1:500 | CST | 3033 |
| IκBα | 1:1000 | CST | 4814 |
| phospho-IκBα | 1:500 | CST | 2859 |
| Total-PI3K | 1:1000 | CST | 4255 |
| Phospho-PI3K | 1:500 | CST | 17366 |
| Total-AKT | 1:1000 | CST | 4685 |
| phospho-AKT (Ser473) | 1:500 | CST | 4060 |
| Histon H3 | 1:1000 | Beyotime | AF0009 |
| β-actin | 1:5000 | Beyotime | AF0003 |
